# Quality of Life and Psychosocial Outcomes Among Children With Cleft Differences

**DOI:** 10.7759/cureus.69176

**Published:** 2024-09-11

**Authors:** Grace Materne, Emily R Disler, Andrew R Scott, Alexander P Marston

**Affiliations:** 1 Otolaryngology - Head and Neck Surgery, Tufts University School of Medicine, Boston, USA; 2 General Surgery, Lahey Hospital & Medical Center, Burlington, USA; 3 Dr. Elie E. Rebeiz Department of Otolaryngology - Head and Neck Surgery, Tufts Medical Center, Boston, USA; 4 Otolaryngology - Head and Neck Surgery, Massachusetts Eye and Ear, Boston, USA; 5 Pediatric Facial Plastic and Craniofacial Surgery, Massachusetts Eye and Ear, Boston, USA; 6 Otolaryngology - Head and Neck Surgery, University of California Davis Health System, Sacramento, USA

**Keywords:** cleft lip, cleft lip and palate, cleft palate, pediatric symptom checklist, psychosocial outcomes, speech, velopharyngeal insufficiency effects on life outcomes

## Abstract

Introduction

Velopharyngeal insufficiency (VPI) and craniofacial differences can lead to diminished speech and swallowing function resulting in communication and social challenges throughout childhood. To monitor changes in patients’ psychosocial health and velopharyngeal function, the Pediatric Symptom Checklist (PSC) and Velopharyngeal Insufficiency Effects on Life Outcomes (VELO) survey tools can be utilized. This study aimed to investigate the relationship between VPI quality-of-life outcomes and psychosocial disturbances through a comparative analysis of PSC and VELO parental surveys among children followed by a craniofacial team.

Methods

A retrospective chart review was completed using data from a single, multidisciplinary cleft and craniofacial team. Previously completed parental survey responses between 2010 and 2022 were collated and results were analyzed using a Spearman's rank correlation test (r_s_).

Results

There were 89 subjects who completed both surveys on the same day (n = 148 survey pairs (s)). Patients aged three to five years old (s = 88) had a mean VELO of 17.9 (0-65) and a mean PSC of 7.9 (0-27), while patients aged six to eight years old (s = 60) had a mean VELO of 16.6 (0-74) and a mean PSC of 12.0 (0-37). The strongest correlation observed for both age groups was between the total PSC and VELO Speech Limitations sub-scores (three to five years old: r_s _= 0.537, p < 0.001; six to eight years old: r_s_ = 0.330, p = 0.010). Similarly, children in the six- to eight-year-old group with cleft lip and palate showed a correlation between the total PSC and VELO Speech Limitations (r_s _= 0.583, p < 0.001).

Conclusion

This study suggests a relationship between PSC and VELO scores among children ages three to eight years old with cleft differences and demonstrates that specific domains within the VELO questionnaire should be considered as being associated with a higher risk for psychosocial impairment. Specifically, higher VELO Speech Limitations sub-scores may portend a greater risk for poor psychosocial outcomes supporting the importance of early interventions in this group.

## Introduction

Children born with cleft and craniofacial differences face challenges that include, but are not limited to, impaired speech and swallowing function, differences in physical appearance, and the need for numerous reconstructive surgeries/interventions, thereby impacting their daily lives. This patient population may experience higher levels of anxiety, negative peer interactions, delayed learning, and diminished self-esteem [1-3]. Several research studies have investigated the connection between velopharyngeal insufficiency (VPI)/craniofacial differences and psychosocial outcomes by administering surveys to patients and their caregivers. In general, children who undergo multiple surgeries early in life are more likely to have higher degrees of psychosocial disturbances [4]. Many pediatric patients with cleft differences will need multiple reconstructive surgeries to restore structure and improve function. Potemra et al. found adolescents who have undergone multiple cleft palate/lip operations report higher rates of long-term depression and anxiety compared to unaffected individuals [5]. Furthermore, De Leon et al. found these differences in psychosocial outcomes (anxiety, peer relationships, and depression) to be more pronounced in patients with craniofacial anomalies, whose parents had limited English proficiency [6]. These studies support the necessity of a multidisciplinary team in the care of patients with cleft differences and the importance of psychosocial evaluation with accessibility of therapy and psychiatric services, when necessary.

Due to the potential negative psychological impact of VPI and craniofacial differences in pediatric patients, several screening surveys are used for routine evaluation and monitoring. The Velopharyngeal Insufficiency Effects on Life Outcomes (VELO) and Pediatric Symptom Checklist (PSC) surveys are both evidence-based screening tools used to assess quality-of-life measures and psychosocial disturbances in children [7,8]. VELO is a quality-of-life survey specific to pediatric patients with VPI that aims to assess how patients’ speech and swallowing function affect their daily lives. The VELO youth survey is completed by the patient (if ≥8 years old) and consists of 23 questions divided into five main sub-scores: Speech Limitations (SL), Swallowing Problems (SP), Situational Difficulty (SD), Emotional Impact (EI), and Perception by Others (PO). The VELO parental survey has 26 questions, with an additional Caregiver Impact (CI) sub-score. Each question is answered using a five-point Likert scale of never (0) to almost always (4) [7]. Assessment of VELO has shown internal consistency and test-retest reliability among multiple cohorts of pediatric patients with VPI [9].

PSC is another instrument used to assess cognitive, emotional, and behavioral problems in pediatric populations and helps inform whether a patient may benefit from psychosocial interventions. PSC is routinely used in assessment and treatment in all pediatric medical specialties. Like the VELO survey, PSC also includes “youth” and “parental” surveys. Both surveys consist of questions assessed via a three-point Likert scale ranging from never (0) to often (2) [8]. For children ages three to five years old, the survey excludes school-related questions, yielding 31 questions on the survey for a maximum potential score of 62 points. In children ages three to five years old, PSC parental survey scores of 24 points or above are considered “at-risk." For children ages six to 17 years old, the school-related questions are included, resulting in a total of 35 questions for a maximum potential score of 70 points and an “at-risk” score of 28 points.

PSC is a validated survey tool that is routinely used in the assessment and treatment of pediatric patients [10,11]. In a National Feasibility Study conducted in 1999, PSC parental surveys were used for psychosocial screening in children across North America (n = 21,065). Overall, the mean parental PSC score for children aged four to 15 years old was 15.1, with 10% (N = 580) of preschool-aged children (four to five years old, n = 5,573) and 13% (N = 2,077) of school-aged children (six to 15 years old, n = 15,492) screening positive for “psychosocial dysfunction” [8]. Furthermore, PSC scores were significantly higher in children from lower socioeconomic status and single-parent households, as well as children with a history of chronic disease or mental health treatment [8,12-14]. This study emphasizes the importance of considering demographic factors while analyzing survey results.

While VELO and PSC are routinely performed in pediatric patients, there are no published data comparing the survey outcomes among pediatric patients with an orofacial cleft. This research study aimed to investigate the quality-of-life and psychosocial disturbances of pediatric patients with cleft differences through a comparative analysis of outcomes reported on VELO and PSC surveys.

This article was previously presented as a poster at the 2023 Combined Otolaryngology Spring Meeting (COSM) as part of the American Society of Pediatric Otolaryngology Annual Meeting on May 5, 2023.

## Materials and methods

A retrospective chart review was completed using data from a single, multidisciplinary cleft and craniofacial team. VELO and PSC surveys previously completed between 2010 and 2022 were collected for pediatric patients with cleft differences (see Appendix A and B). These surveys were completed by patients or their caregivers during initial and follow-up appointments with the cleft and craniofacial team. A VELO youth survey was completed by the patient if they were older than eight years old, while a PSC youth survey was completed once the patient was >10 years old. For younger children, their caregivers completed the parent version of each survey. Paper copies of the surveys were provided, and instructions were given in both oral and written form by the care team. After completion, all paper surveys were scanned into the patient’s chart, and total survey scores were documented in the patient’s visit notes. Measures to maintain survey accuracy included using the same survey forms/instructions, longitudinal tracking of data, and frequent documentation. Given the retrospective nature of the study, we were unable to control for other factors such as the specific caregiver completing the survey or the environment of survey administration.

All available parental and youth surveys were collected for patients with cleft lip (CL), cleft palate (CP), and cleft lip and palate (CLP). Additional patient information, such as age, sex, race, ethnicity, insurance status, cleft type, and syndromic status, was collected from the patient's electronic medical record (EMR). All patient data was de-identified, and each subject was given a coded number. All data were entered and stored in REDCap and analyzed using Excel (v16.52, Microsoft Corporation, USA) and IBM SPSS Statistics for Windows, (v28.0.1.1, IBM Corp., Armonk, NY). This study was approved by the Tufts Health Sciences Institutional Review Board at Tufts Medical Center (approval no. STUDY00002637). Given the retrospective survey design of the study, the IRB waived the requirement for informed consent.

**Figure 1:**
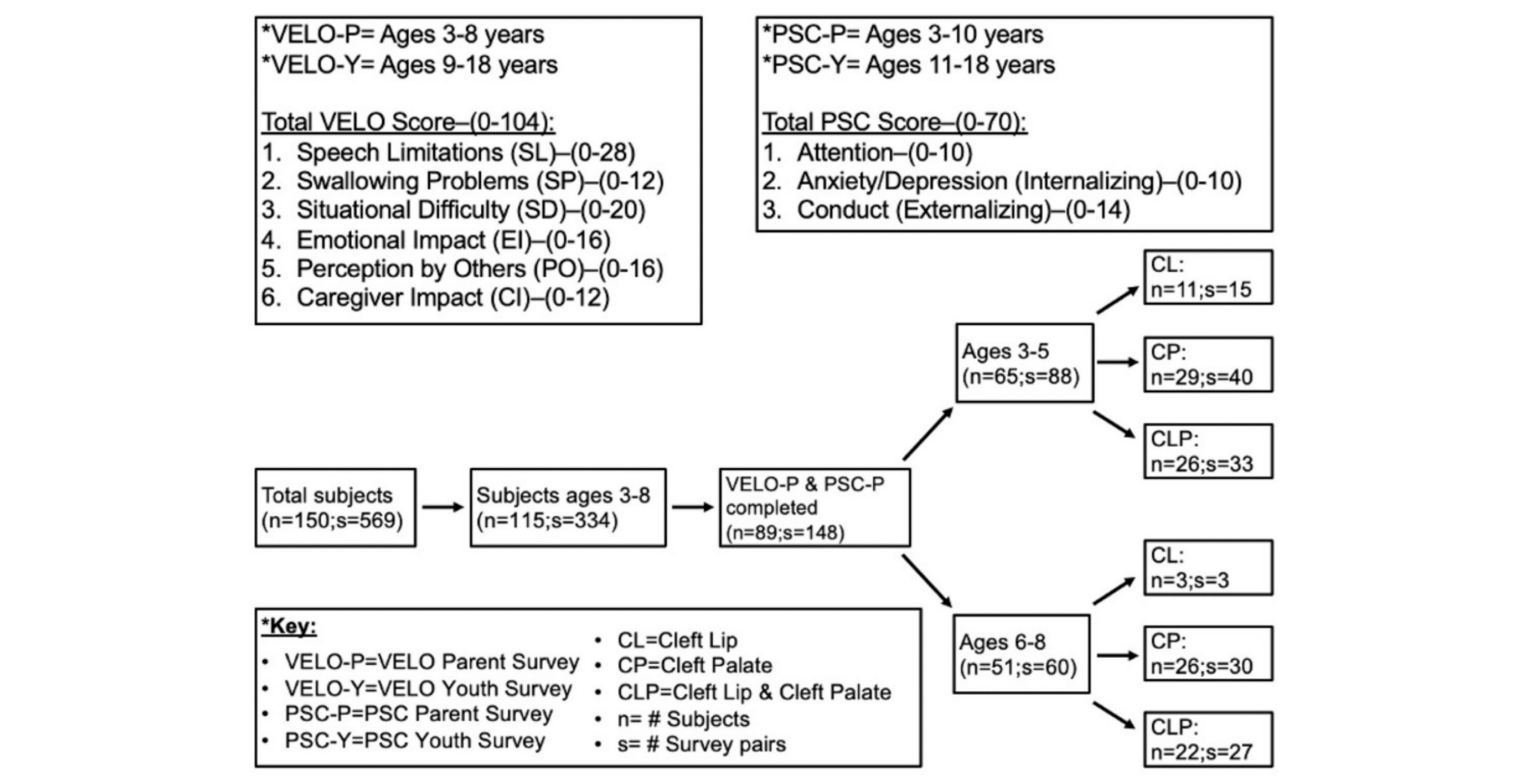
Summary of filtering methods to form the patient population for comparative analysis The final study population consisted of 148 survey pairs (s), representing 89 individual patients (n).

Per the PSC scoring guidelines, data were filtered to exclude surveys with four or more unanswered questions. PSC and VELO youth surveys were also excluded from the analysis due to the lower survey numbers and variable scoring criteria compared to the parental surveys. Subjects were divided into two age groups: children aged three to five years old and six to eight years old. In addition, to complete a comparative analysis between VELO and PSC, the subjects were filtered to capture patients ages three to eight years old who had a VELO and a PSC parental survey (a survey pair (s)) completed on the same day. For data analysis purposes, each survey pair was considered independent even if they were completed for the same patient at different time points; thus, there were more survey pairs (s) than total patients (n) in the study. Analysis was performed by age groups to control for PSC survey questions pertaining to a child’s school experience. When the PSC results were compared between age groups, school-related questions were not included in the total score calculation. The parental survey scores were then stratified by VELO survey sub-scores (Figure 1). Due to the unequal variance in the data, non-parametric statistical tests were used to complete a comparative analysis between PSC and VELO. Mann-Whitney U tests were used to compare mean survey scores between age and cleft groups. A Spearman’s rank correlation coefficient test (r_s_) was performed to measure the correlation between VELO total and sub-scores versus PSC total scores based on age and cleft type. Additional correlation tests were performed comparing parental survey results based on cleft type (CL, CP, CLP), insurance status, sex, and syndromic status. The statistical significance of correlation coefficients between sub-groups within each age group was then calculated.

## Results

There was a total of 569 survey instances (VELO, PSC, or VELO and PSC) completed by/for 150 pediatric patients with cleft differences between 2010 and 2022. The data were filtered to meet the constraints of the intended data analysis (Figure 1). The final study population consisted of 89 pediatric patients with a total of 148 survey pairs due to several patients completing multiple surveys during the study period. Table 1 highlights the age distribution, cleft types, and demographics of the study population.

**Table 1:** Demographics of the patient population The data are represented as s and %. There were a total of 148 survey pairs (s). s: survey pairs Race and ethnicity documented as "Other" was not defined by the electronic medical record.

	s	%
Age		
3-5 years old	88	59.5%
6-8 years old	60	40.5%
Sex		
Male	92	62.2%
Female	56	37.8%
Race		
Asian	13	8.8%
Black	7	4.7%
Hispanic	32	21.6%
White	91	61.5%
Other	5	3.4%
Cleft type		
Cleft lip	18	12.2%
Cleft palate	70	47.3%
Cleft lip and palate	60	40.5%
Syndrome	49	33.1%
Insurance		
Public	78	52.7%
Private	70	47.3%

Table 2 displays the mean PSC and VELO scores for each age group, stratified by the cleft type. In addition, Table 2 highlights the considerable variance and spread among survey scores, suggestive of non-normal data distribution. PSC scores were significantly higher in six to eight years old compared to three to five years old (12.0 versus 7.9, p = 0.018). While the mean VELO scores between the two age groups were not statistically significant, when the data were stratified by cleft type, children with CLP had significantly higher PSC and VELO total/sub-scores compared to children with CL and CP. This relationship was especially evident in the younger three to five-year-old age group (Table 3A).

**Table 2:** Summary statistics of the PSC and VELO total survey scores among subjects stratified by age group and cleft type VELO and PSC were completed on the same day. The survey score data are represented as s, mean PSC/VELO score, standard deviation, and range (minimum-maximum). The data are further stratified by age group and cleft type. s: survey pairs; x̄: mean; SD: standard deviation, CL: cleft lip, CP: cleft palate, CLP: cleft lip and palate

		PSC			VELO		
	s	x̄	SD	Range	x̄	SD	Range
3-5 years old	88	7.9	6.6	0-28	17.9	16.0	0-65
CL	15	6.7	5.6	0-19	8.9	11.2	0-31
CP	40	6.0	5.6	0-20	16.3	15.9	0-51
CLP	33	10.8	7.2	0-28	23.9	16.0	0-65
6-8 years old	60	12.0	9.3	0-37	16.6	14.6	0-74
CL	3	6.0	5.3	0-10	20.3	31.8	2-57
CP	30	11.5	8.9	0-37	14.1	9.3	0-27
CLP	27	13.1	9.9	0-37	19.0	17.3	0-74

**Table 3:** VELO and PSC mean scores and Spearman's rank correlation in patients ages three to five years old (A) and six to eight years old (B) The survey score data are represented as mean VELO/PSC score and Spearman's rank correlation coefficient (r_s_) between the VELO total and sub-scores versus PSC total score. The data are further stratified by age group and cleft type. Arrows indicate whether the mean survey score for a specific cleft type was above or below the overall mean score. s: survey pairs; x̄: mean; r_s_: Spearman's rank correlation coefficient CL: cleft lip, CP: cleft palate, CLP: cleft lip and palate, SL: Speech Limitations, SD: Situational Difficulty, EI: Emotional Impact, Statistical significance (p ≤ 0.05) between means is denoted as superscripts. ^CL^: significant difference when compared to patients with cleft lip, ^CP^: significant difference when compared to patients with cleft palate, ^CLP^: significant difference when compared to patients with cleft lip and palate

A.	VELO (x̄)		PSC (x̄)		r_s_	p (r_s_)
Total VELO (0-104)						
All (s=88)	17.9	––	7.9	––	0.529	<0.001
CL (s=15)	8.9^CLP^	↓	6.6^CLP^	↓	0.814^CP, CLP^	<0.001
CP (s=40)	16.3^CLP^	↓	5.9^CLP^	↓	0.513^CL^	<0.001
CLP (s=33)	23.9^ CL, CP^	↑	10.7^CL, CP^	↑	0.404^CL^	0.020
SL (0-28)						
All	6.9	––	7.9	––	0.537	<0.001
CL	3.9^ CLP^	↓	6.6^ CLP^	↓	0.762	<0.001
CP	6.2^CLP^	↓	5.9^ CLP^	↓	0.457	0.003
CLP	9.0^CL, CP^	↑	10.7^ CL, CP^	↑	0.462	0.007
SD (0-20)						
All	5.4^ CLP^	––	7.9	––	0.471	<0.001
CL	2.3	↓	6.6^ CLP^	↓	0.830^ CP, CLP^	<0.001
CP	4.9^CLP^	↓	5.9^ CLP^	↓	0.434^ CL^	0.005
CLP	7.6^ CL, CP^	↑	10.7^ CL, CP^	↑	0.335^ CL^	0.056
EI (0-16)						
All	1.8	––	7.9	––	0.524	<0.001
CL	1.1	↓	6.6^ CLP^	↓	0.628	0.012
CP	1.7	↓	5.9^ CLP^	↓	0.554	<0.001
CLP	2.2	↑	10.7^ CL, CP^	↑	0.407	0.019

B.	VELO (x̄)		PSC (x̄)		r_s_	p (r_s_)
Total VELO (0-104)						
All (s=60)	16.6	––	12.0	––	0.295	0.022
CP (s=30)	14.1	↓	11.5	↓	-0.088 ^CLP^	0.644
CLP (s=27)	19.0	↑	13.1	↑	0.629^CP^	<0.001
SL (0-28)						
All	6.8	––	12.0	––	0.330	0.01
CP	6.3	↓	11.5	↓	0.042 ^CLP^	0.825
CLP	7.3	↑	13.1	↑	0.583^ CP^	0.001
SD (0-20)						
All	4.8	––	12.0	––	0.263	0.042
CP	4.4	↓	11.5	↓	-0.065 ^CLP^	0.733
CLP	5.4	↑	13.1	↑	0.547^ CP^	0.003
EI (0-16)						
All	1.9	––	12.0	––	0.282	0.029
CP	1.1	↓	11.5	↓	0.040^ CLP^	0.833
CLP	2.5	↑	13.1	↑	0.498^ CP^	0.008

**Figure 2:**
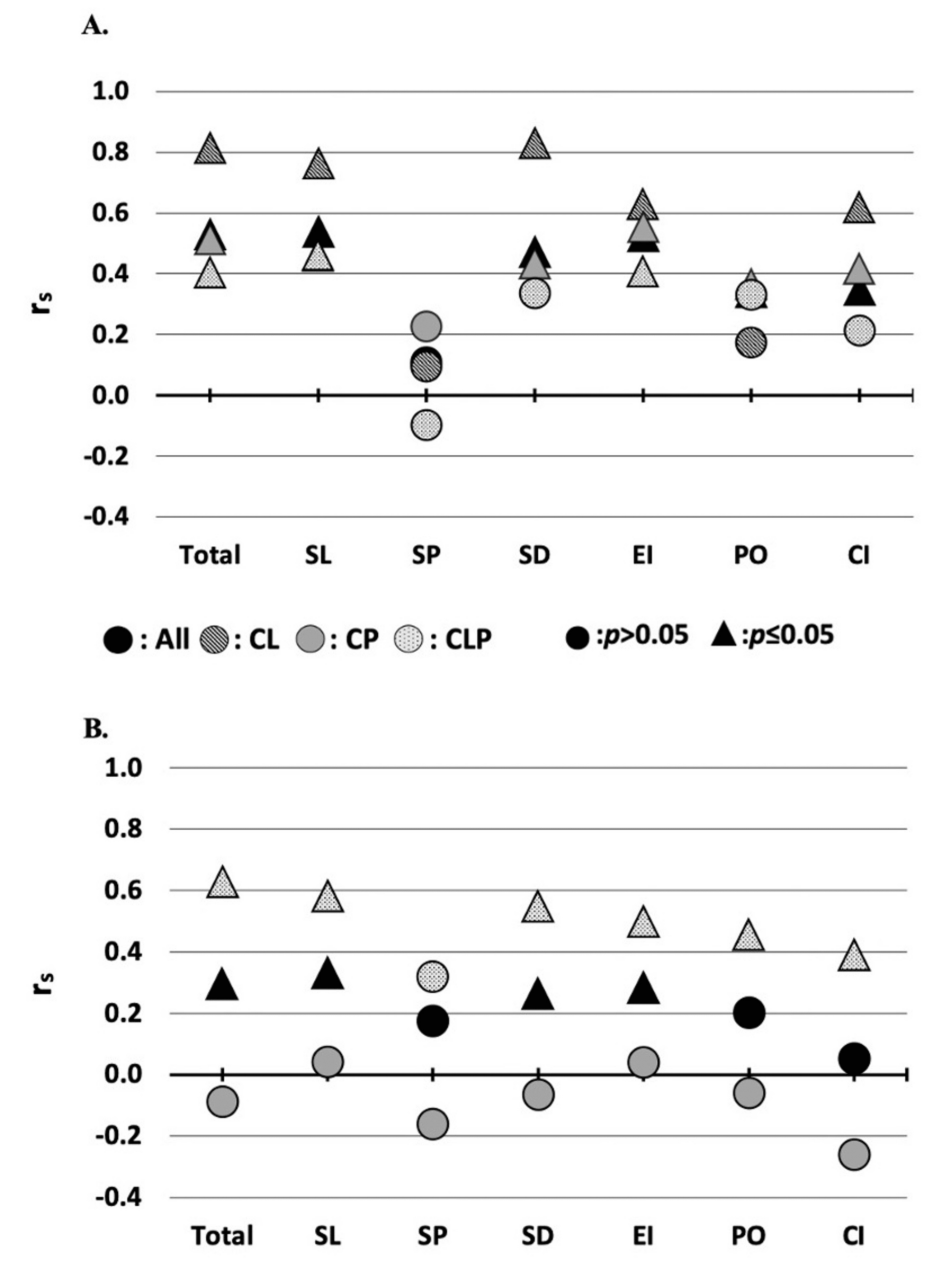
Spearman's rank correlation of the total PSC score versus VELO scores in patients ages three to five years old (A) and six to eight years old (B) The data are represented as Spearman's rank correlation coefficient between the VELO total and sub-scores versus the PSC total score. The data are further stratified by age group and cleft type. Statistical significance (p ≤ 0.05) is represented by the shape of the data point. The number of patients with a cleft lip in the older age group was inadequate to complete the analysis. r_s_: Spearman's rank correlation coefficient, CL: cleft lip, CP: cleft palate, CLP: cleft lip and palate, SL: Speech Limitations, SP: Swallowing Problems, SD: Situational Difficulty, EI: Emotional Impact, PO: Perception by Others, CI: Caregiver Impact

The Spearman’s rank correlation results are demonstrated in Figures 2A-2B. There was a statistically significant correlation between the total VELO and total PSC scores for both age groups; however, patients ages three to five years old (r_s _= 0.529, p < 0.001) had a stronger correlation compared to patients ages six to eight years old (r_s_ = 0.295, p ≤ 0.05). Among VELO sub-scores in both age groups, significant correlations were observed in the SL, SD, and EI categories. In patients ages three to five years old, significant correlations were also observed in CI and PO categories (Figures 2A, 2B). In the younger age group, the strongest correlation was between the VELO SL sub-score and total PSC score (r_s_ = 0.537), while strong correlations were also observed in the EI (r_s_ = 0.524) and SD (r_s _= 0.471) sub-scores (Table 3A). Similarly, for patients ages six to eight years, the strongest correlation was between the VELO SL sub-score versus the total PSC score (r_s_ = 0.330), while statistically significant correlations (p ≤ 0.05) were also observed in the EI (r_s_ = 0.282) and SD (r_s_ = 0.263) sub-scores (Table 3B). Comparing the VELO sub-scores between the age groups, the EI and CI correlations were significantly stronger in the younger age group (p ≤ 0.05).

When stratified by the cleft type, the mean VELO and PSC scores for the CL and CP groups were consistently lower compared to patients with CLP and the overall group (Table 3A). Of note, older patients with CLP had the strongest correlations (p ≤ 0.05) across the VELO categories and the highest mean VELO and PSC scores in their group (although this difference in mean scores was not statistically significant) (Table 3B). Regardless, both age groups demonstrated a consistent directional trend between the total VELO and PSC scores, represented by the correlation coefficient (Table 3). In addition, all cleft types in both age groups (except CP in the six- to eight-year-old group), exhibit strong, statistically significant (p ≤ 0.05) correlations between the PSC score and VELO sub-category scores representing the SL, SD, and EI categories.

**Figure 3:**
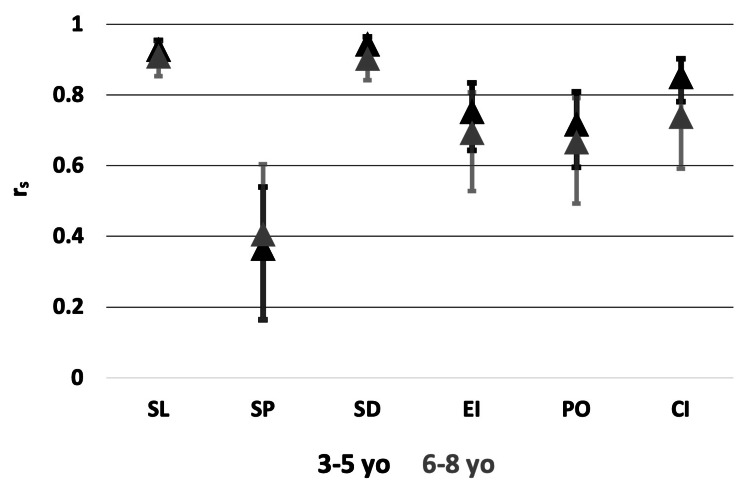
Spearman's rank correlation of the total VELO score versus VELO sub-scores by age group The data are represented as Spearman's rank correlation coefficient between the VELO total versus VELO sub-scores. The data are further stratified by age group. Error bars represent data range (minimum, maximum). r_s_: Spearman's rank correlation coefficient, SL: Speech Limitations, SP: Swallowing Problems, SD: Situational Difficulty, EI: Emotional Impact, PO: Perception by Others, CI: Caregiver Impact, yo: years-old

Total VELO scores are typically included in the patients’ charts as a part of the multidisciplinary cleft team documentation; however, the individual VELO sub-scores are seldom recorded. To study how specific VELO sub-scores contribute to the total VELO score, an additional Spearman’s rank correlation coefficient test was conducted. Figure 3 highlights how the SL and SD sub-scores contribute the greatest to the total VELO score compared to the other sub-scores across both age groups (p ≤ 0.05).

The correlation between the PSC and VELO total scores was further assessed via stratification by race, sex, syndromic status, and insurance type (Tables 4A, 4B). Race stratification in the younger age group revealed the highest PSC and VELO scores in Hispanic patients and the greatest correlation coefficients in Black and Asian subjects. In the older age group, Hispanic patients had significantly higher mean PSC scores compared to White patients (p ≤ 0.05). Due to the considerable variation in racial distribution, conclusions regarding the impact of race on survey scores cannot be drawn from this data. Furthermore, when patients aged three to five years old were stratified by sex, male patients had higher mean PSC scores compared to females (p ≤ 0.05). When stratified by insurance status, older patients with public insurance had significantly (p ≤ 0.05) higher PSC scores compared to those with private insurance (Table 4B).

**Table 4:** Summary statistics stratified by demographic characteristics for the three- to five-year-old age group (A) and six- to eight-year-old age group (B) The data are represented as s, mean PSC/VELO scores, Spearman's rank correlation coefficient between the VELO total score versus PSC total score and 95% confidence interval. The data are further stratified by age group and patient demographics. s: survey pairs; x̄: mean; r_s_: Spearman's rank correlation coefficient; CI: confidence interval Statistical significance (p ≤ 0.05) between means is denoted as superscripts: *: significant difference between groups, ^A^: significant difference when compared to patients who identify as Asian, ^B^: significant difference when compared to patients who identify as Black, ^H^: significant difference when compared to patients who identify as Hispanic, ^W^: significant difference when compared to patients who identify as White, ^O^: significant difference when compared to patients who identify as "Other" Race and ethnicity documented as "Other" was not defined by the electronic medical record.

A.	s	PSC (x̄)	VELO (x̄)	r_s_	p (r_s_)	95% CI	
Sex							
Male	53	8.8*	19.4	0.5457	<0.001	0.2	0.7
Female	35	6.6*	15.7	0.585	<0.001	0.3	0.8
Race							
Asian	11	9.4	17.0^B^	0.829^H,W^	0.002	0.4	1.0
Black	5	4.2^H^	1.6^A,H,W^	0.918^H^	0.028	0.1	1.0
Hispanic	15	11.2^B,O^	21.5^B,O^	0.270^A,B^	0.331	-0.3	0.7
White	53	7.5^O^	19.4^B^	0.513^A^	<0.001	0.3	0.7
Other	4	1.8^W,H^	7.8^H^	0.211	0.789	-0.9	1.0
Syndrome							
Yes	28	7.0	20.6	0.422	0.025	0.04	0.7
No	60	8.3	16.7	0.663	<0.001	0.5	0.8
Insurance type							
Public	39	9.3	18.7	0.567	<0.001	0.3	0.8
Private	49	6.8	17.3	0.478	<0.001	0.2	0.7

B.	s	PSC (x̄)	VELO (x̄)	r_s_	p (r_s_)	95% CI	
Sex							
Male	39	13.5	17.6	0.340	0.034	0.02	0.6
Female	21	9.1	14.8	0.155	0.503	-0.3	0.6
Race							
Asian	2	4.5	42.0^W^	NA	NA	NA	NA
Black	2	0.5^H,W^	1.0^H,W^	NA	NA	NA	NA
Hispanic	17	17.1^B,W^	19.3^B^	0.262	0.31	-0.3	0.7
White	38	10.8^B,H^	15.1^A,B^	0.340	0.037	0.01	0.6
Other	1	8.0	9.0	NA	NA	NA	NA
Syndrome							
Yes	21	14.3	15.6	-0.022*	0.925	-0.5	0.4
No	39	10.7	17.1	0.421*	0.008	0.1	0.7
Insurance type							
Public	31	15.1*	16.5	0.246	0.183	-0.1	0.6
Private	29	8.7*	16.7	0.397	0.033	0.03	0.7

## Discussion

VELO is a well-accepted quality-of-life questionnaire to screen and describe the severity of velopharyngeal insufficiency among craniofacial teams; however, potential relationships between the VELO and other psychosocial survey instruments, such as the PSC, are not well-described in the literature [7,15]. In this study, higher PSC scores found in patients ages six to eight years old suggest poorer psychosocial outcomes in this age group compared to younger patients. This result may be due to increased social pressures from peers and heightened self-awareness in school-aged children [5,16-20]. Among children with clefts, there is a higher rate of teasing reported in older children and adolescents, which negatively impacts their socioemotional health and peer interactions [16,21,22]. Furthermore, when patients aged 16-25 years old with cleft differences and their parents were interviewed, 25% (N = 7) of patients reported that they were teased as children, while over 50% (N ≥ 14) of patients stated that their cleft negatively impacted their self-confidence and ability to form intimate relationships [23]. In addition, when the patient’s parents were interviewed, over half believed their child’s cleft difference adversely impacted their emotional and social health, including their school performance and ability to make friends [23]. When patients aged 15-20 years old were surveyed, 73% (N = 38) of patients reported significantly reduced self-esteem due to their cleft differences and subsequent teasing from peers about their speech [19]. The findings presented in our study align with existing research and published data, reinforcing the importance of early mental health screening and intervention among children with cleft differences.

The correlation between VELO and PSC scores suggests a relationship between functional impairments and psychosocial health among patients with cleft differences. For instance, consistently higher VELO and PSC scores for children with CP and CLP suggest that this population may experience higher rates of negative psychosocial effects and diminished quality of life throughout childhood compared to other cleft types. Another retrospective survey study that analyzed quality-of-life outcomes in patients with cleft differences found lower parent-reported quality-of-life scores in children with CP compared to CL [24]. This finding may be due to the impact of a CP on speech disturbance and the potential for more frequent surgeries required for patients with CP and CLP compared to those with isolated CL, contributing to lower parent scoring of their child’s social function and engagement in activities [24,25]. When interpreting higher VELO scores in younger patients (three to five years old), it is important to consider that these children are likely not enrolled in school and may not have access to speech therapy or special education services yet; thus, their speech differences are more noticeable to their caregivers. Conversely, patients with more visible cleft differences (CL and CLP) report higher rates of stigma and dissatisfaction with their appearance compared to patients with isolated CP [24,26]. This finding may help explain higher PSC surveys in patients with CLP given that these patients may have more noticeable differences and scars compared to patients with isolated CL or CP.

This study highlights not only the correlation between PSC and total VELO surveys in pediatric patients but also between PSC and VELO sub-scores. This relationship indicates that specific functional impairments attributed to a patient’s cleft may have a more prominent impact on their overall psychosocial status. Of note, the SL, SD, and EI VELO sub-scores had the greatest overall effect on patients’ self-reported mental health assessment across cleft types. Furthermore, the data demonstrate that specifically patients’ SL VELO sub-scores were highly correlated with their psychosocial assessment (PSC). When stratified by cleft type, patients with CLP in both age groups exhibited significantly higher VELO total scores and sub-scores. Previous studies have shown a strong connection between speech and quality-of-life outcomes in the cleft population [18,19,26-28]. For instance, Bhuskute et al. demonstrated a correlation between enhancements in speech intelligibility and improvements in the VELO total and sub-scores by studying survey results before and after patients underwent cleft repair [12]. The results of our study not only support this existing relationship but also suggest that patients with CP may experience a worse quality of life due to speech disturbances. Similarly, a prospective cohort study by Zeraatkar et al. found the greatest concern of children with CLP aged four to six years old and their parents was impaired speech [28]. Children’s speech contributed to poor socioemotional well-being, including heightened feelings of shame, anxiety, and fewer peer interactions [28]. Therefore, implementing early speech therapy and/or additional treatment focused on speech for children with cleft differences may be able to improve psychosocial outcomes over time. 

In addition, individual VELO sub-scores were correlated to the total VELO score; thus, sub-scores, including SL and SD, may be predictive of the VELO survey results. The SL and SD sub-scores specifically assess functional speech impairments, while the other VELO sub-scores focus on the emotional consequences of the child’s speech/swallowing function. These results support the need to assess patients for potential surgical and therapeutic interventions addressing speech concerns. In addition, this analysis reveals the importance of considering and documenting one’s overall and categorical VELO scores to help determine the need for psychiatric referral. 

It is important to consider VELO and PSC results in the context of underlying demographic factors. Due to the small sample size and unequal variance across racial and socioeconomic status (SES) groups, it is difficult to assess the generalizability of this study. While PSC has been studied in diverse populations, there is limited data on variability in VELO results based on sex, race, or SES. Prior studies have found that male sex and lower SES are associated with higher PSC scores [8]. These patterns were also uncovered in our project, with male patients and patients with public insurance having higher PSC scores. Furthermore, racial and financial inequities are apparent in the cleft population. In one study, children with higher SES and those who identified as White had lower rates of cleft-associated stigma and dissatisfaction with appearance, as well as improved speech outcomes [24]. Equitable access to early speech and psychosocial interventions is essential to promote future development and successful integration into society [24,27]. 

Study limitations include the small sample size with unequal variance across demographic subgroups and cleft types, especially among isolated CL patients. In addition, there is no record of patients’ mental health or history of past psychiatric treatment. Moreover, the retrospective nature of the study introduces bias in survey completion, such as the environment in which the survey was completed and whether the same caregiver completed the survey each time. External factors like the education of the caregiver completing the survey, the relationship of the caregiver to the child (biological parent versus guardian), the number of caregivers at home, and the caregiver’s mental health or anxiety could all influence the survey responses. Furthermore, the significant spread in the data restricted the analysis to non-parametric statistical tests and limited evaluation of possible confounding variables. Thus, it is difficult to assess how representative the study sample is of the overall population of patients with cleft differences.

Further research is needed to determine longitudinal trends in VELO and PSC surveys and investigate possible confounding variables influencing survey results. For instance, this study did not consider how survey scores changed after patients underwent surgery or other treatments for their craniofacial differences. Analyzing the effect of surgery on VELO and PSC scores could help inform postoperative psychosocial outcomes and potentially influence future treatment decisions. In addition, it would be helpful to include assessments completed by subjects’ daycare and/or schoolteachers since they witness children’s cognitive and social development daily and can compare it to the level of their peers. In fact, Murray et al. found teacher report forms revealed significant differences in the evaluation of a child’s socialization compared to parent surveys [29].

## Conclusions

This retrospective survey study was completed to assess the relationship between quality-of-life and psychosocial outcomes among patients with cleft differences. The analyzed data suggest that functional impairments captured on the VELO questionnaire are likely critical predictive factors for poor psychosocial status as demonstrated by the PSC survey scores among children with cleft differences. As a result of this study’s findings, it may be important to consider a patient’s overall and categorical VELO scores when assessing the need for mental health care resources and psychiatric referrals. Specifically, higher VELO sub-scores in Speech Limitations, Situational Difficulty, and Emotional Impact may help to identify a patient in need of further psychosocial evaluation that otherwise may screen negative. In addition, this study highlights how additional speech-focused early interventions could improve patients’ emotional well-being. Overall, the application of these findings supports the use of both PSC and VELO surveys in clinical practice when caring for patients with cleft differences.

## Appendices

Appendix A

**Table 5:** Pediatric Symptom Checklist (PSC) survey tool Pediatric Symptom Checklist (PSC) survey tool developed by Jellinek et al., 1988 [10].

		Never (0)	Sometimes (1)	Often (2)
1	Complains of aches and pains			
2	Spends more time alone			
3	Tires easily, has little energy			
4	Fidgety, unable to sit still			
5	Has trouble with teacher			
6	Less interested in school			
7	Acts as if driven by a motor			
8	Daydreams too much			
9	Distracted easily			
10	Is afraid of new situations			
11	Feels sad, unhappy			
12	Is irritable, angry			
13	Feels hopeless			
14	Has trouble concentrating			
15	Less interested in friends			
16	Fights with other children			
17	Absent from school			
18	School grades dropping			
19	Is down on him or herself			
20	Visits doctor with doctor finding nothing wrong			
21	Has trouble sleeping			
22	Worries a lot			
23	Wants to be with you more than before			
24	Feels he or she is bad			
25	Takes unnecessary risks			
26	Gets hurt frequently			
27	Seems to be having less fun			
28	Acts younger than children his or her age			
29	Does not listen to rules			
30	Does not show feelings			
31	Does not understand other people’s feelings			
32	Teases others			
33	Blames others for his or her troubles			
34	Takes things that do not belong to him or her			
35	Refuses to share			

Appendix B

**Table 6:** Velopharyngeal Insufficiency Effects on Life Outcomes (VELO) survey tool Velopharyngeal Insufficiency Effects on Life Outcomes (VELO) survey tool developed by Skirko et al., 2012 [7].

		Never (0)	Almost never (1)	Sometimes (2)	Often (3)	Almost always (4)
Speech Limitations (problems with…)						
1	Air comes out his or her nose when talking					
2	Runs out of breath when talking					
3	Difficulty speaking in long sentences					
4	Speech is too weak					
5	Difficulty being understood when in a hurry					
6	Speech gets worse toward the end of the day					
7	Speech sounds different than other kids					
Swallowing Problems (problems with...)						
8	Liquids come from the nose while drinking					
9	Solid food comes from the nose while eating					
10	Others make fun of my child when food or liquids escape through the nose					
Situational Difficulty (problems with...)						
11	Speech is difficult for strangers to understand					
12	Speech is difficult for friends to understand					
13	Speech is difficult for family to understand					
14	Difficulty being understood when not speaking face to face, eg, as in a car					
15	Difficulty being understood on the phone					
Emotional Impact (problems with...)						
16	Teased because of speech					
17	Child gets sad because of speech					
18	Gets frustrated or gives up when he or she is not understood					
19	Is shy or withdrawn because of speech					
Perception by Others (problems with...)						
20	Treated as if he or she is not very bright because of speech					
21	Others ignore my child because of his or her speech					
22	Others do not like to talk on the phone with my child because of his or her speech					
23	Family or friends tend to speak for my child					
Caregiver Impact (problems with...)						
24	I am worried or concerned about my child’s speech					
25	I find it difficult to understand my child					
26	My child’s speech problem slows me down or inconveniences me					
